# *U2AF1* pathogenic variants in myeloid neoplasms and precursor states: distribution of co-mutations and prognostic heterogeneity

**DOI:** 10.1038/s41408-023-00922-7

**Published:** 2023-09-21

**Authors:** Talha Badar, Yenny A. Moreno Vanegas, Ahmad Nanaa, James M. Foran, Aref Al-Kali, Abhishek Mangaonkar, Hemant Murthy, Hassan B. Alkhateeb, David Viswanatha, Rong He, Mithun Shah, Cecilia Arana Yi, Mark R. Litzow, Naseema Gangat, Ayalew Tefferi, Mrinal M. Patnaik

**Affiliations:** 1 Division of Hematology-Oncology and Bone Marrow Transplant Program, Mayo Clinic, Jacksonville, FL 32224 USA; 2 Division of Hematology, Mayo Clinic, Rochester, MN 55905 USA; 3 John H. Stroger, Jr. Hospital of Cook County, Chicago, IL 60612 USA; 4 Division of Hematopathology, Mayo Clinic, Rochester, MN 55905 USA; 5 Department of Hematology Oncology, Mayo Clinic, Phoenix, AZ USA

**Keywords:** Myelodysplastic syndrome, Cancer epigenetics

## Abstract

We have previously recognized the genotypic and prognostic heterogeneity of *U2AF1* mutations (MT) in myelofibrosis (MF) and myelodysplastic syndromes (MDS). In the current study, we considered 179 *U2AF1*-mutated patients with clonal cytopenia of undetermined significance (CCUS; *n* = 22), MDS (*n* = 108), MDS/acute myeloid leukemia (AML; *n* = 18) and AML (*n* = 31). *U2AF1* variants included S34 (60%), Q157 (35%), and others (5%): corresponding mutational frequencies were 45%, 55%, and 0% in CCUS; 57%, 39%, and 4% in MDS; 61%, 33%, and 6% in MDS/AML; and 55%, 35% and 10% in AML (*P* = 0.17, 0.36 and 0.09), respectively. Concurrent mutations included *ASXL1* (37%), *BCOR* (19%), *RUNX1* (14%), *TET2* (15%), *DNMT3A* (10%), *NRAS*/*KRAS* (8%), *TP53* (8%), *JAK2* (5.5%) and *SETBP1* (5%). The two most frequent *U2AF1* MT were S34F (*n* = 97) and Q157P (*n* = 46); concurrent MT were more likely to be seen with the latter (91% vs 74%; *P* = 0.01) and abnormal karyotype with the former (70% vs 62%; *P* = 0.05). *U2AF1* S34F MT clustered with *BCOR* (*P* = 0.04) and Q157P MT with *ASXL1* (*P* = 0.01) and *TP53* (*P* = 0.03). The median overall survival (OS) in months was significantly worse in AML (14.2) vs MDS/AML (27.3) vs MDS (33.7; *P* = 0.001); the latter had similar OS with CCUS (30.0). In morphologically high-risk disease (*n* = 49), defined by ≥10% blood or bone marrow blasts (i.e., AML or MDS/AML), median OS was 14.2 with Q157P vs 37.1 months in the presence of S34F (*P* = 0.008); transplant-adjusted multivariable analysis confirmed the detrimental impact of Q157P (*P* = 0.01) on survival and also identified *JAK2* MT as an additional risk factor (*P* = 0.02). OS was favorably affected by allogeneic hematopoietic stem cell transplantation (HR: 0.16, 95% CI; 0.04-0.61, *P* = 0.007). The current study defines the prevalence and co-mutational profiles of *U2AF1* pathogenic variants in AML, MDS/AML, MDS, and CCUS, and suggests prognostic heterogeneity in patients with ≥10% blasts.

## Introduction

Myelodysplastic neoplasm/syndrome (MDS) and acute myeloid leukemia (AML) are heterogeneous diseases, with variable outcomes, largely driven by chromosomal alterations and somatic mutations [1, 2]. Mutations in genes encoding components of the spliceosome complex (*SRSF2*, *U2AF1*, *SF3B1*, *ZRSR2*) are observed in approximately one-third of the patients with MDS and nearly half of the patients with MDS transforming to AML (secondary [s] AML) [3–6]. Historically, AML patients with gene mutations related to spliceosome complex, including *U2AF1* were considered as intermediate risk, but with recently updated European LeukemiaNet (ELN) 2022 recommendations, these patients are now considered adverse risk based on associated inferior outcomes [7]. *U2AF1* is an important component of the spliceosome complex required for pre-mRNA splicing [8, 9]. Mutations in *U2AF1* have been described in myeloid neoplasms, with variants causing specific alterations in 3′ splice site recognition [5, 10, 11]. *U2AF1* mutations (MT) are typically acquired later in life and associated with rapid rates of progression to MDS and AML [12–17] *U2AF1* MT predominantly occur at two hotspots (S34F, Q157) located in zinc finger regions [5]. An earlier report evaluating clinical outcomes of 78 MDS patients with *U2AF1* MT demonstrated that transcriptional factor and epigenetic regulator genes (e.g., *ASXL1* [26%], *DNMT3A/PHF6* [12%], *BCOR* [15%], *TET2* [13%], *RUNX1/STAG2* [9%], *SETBP1*[8%]) were predominantly co-mutated in *U2AF1* MT myeloid neoplasms. Furthermore, analysis showed that ancestral *U2AF1*^S34F^ MT were associated with inferior outcomes in comparison to ancestral *U2AF1*^Q157^ MT, while no differences in outcomes were observed if the mutations were secondary/subclonal in nature [15]. In another study by Tefferi et al. [17], 52 MDS patients with *U2AF1* MT were evaluated, S34F and Q157 hotspots were commonly observed. However, a cytogenetic-independent prognostic impact was not evident for either one of the two commonly observed hotspot mutations. Unlike in MDS, the mutational spectrum of *U2AF1* MT in myelofibrosis is contrastingly different with higher occurrence of Q157 (50/77 [65%]) compared to S34 (26/77 [34%]) hotspots [10]. To the best of our knowledge apart from the aforementioned studies, limited information is available on the molecular profile, myeloid co-mutation pattern and survival outcomes with unique *U2AF1* MT in patients with clonal cytopenia of undetermined significance (CCUS), MDS, MDS/AML and AML. In this report we have analyzed the genomic profile and clinical relevance of *U2AF1* MT in a larger cohort of patients with myeloid neoplasms.

## Patient and methods

We reviewed the Mayo Clinic database of patients with myeloid neoplasms who underwent next-generation genomic sequencing (NGS) between January 2015 and July 2021. We evaluated the molecular profile and outcomes in 179 patients with precursor myeloid neoplasms (clonal cytopenias of undetermined significance [CCUS; *n* = 22]) and myeloid neoplasms (MDS [*n* = 108], MDS/AML [*n* = 18] and AML [*n* = 31]) harboring *U2AF1* MT. Clinical NGS testing was performed on DNA extracted from fresh bone marrow aspirates. The Mayo Clinic NGS panel included 42 genes (Supplementary Material) and has an accuracy of >99% and reproducibility of 100% for single base substitutions and insertion/deletion events. The panel has a variant sensitivity of ≥2% VAF with a minimum depth coverage of 250x. CCUS was defined according to the 2022 WHO (World Health Organization) criteria; MDS, MDS/AML and AML were defined as per International Consensus Classification (ICC) 2022 of myeloid neoplasms and acute leukemia [18, 19]. For this analysis, we operationally defined high-risk myeloid neoplasms as myeloid neoplasms with ≥10% myeloid blasts in the peripheral blood and/or bone marrow. Treatment responses in MDS and AML were assessed according to the International Working Group (IWG) MDS response criteria (2006) and the 2017 ELN AML response criteria, respectively [20, 21].

### Statistical analysis

Continuous variables summarized as medians (range), while categorical variables reported as frequencies (percentage). Unadjusted comparisons of patient characteristics and outcomes among patients with different myeloid neoplasms and *U2AF1* MT were made using the Wilcoxon rank sum test (continuous variables) or Fisher’s exact test (categorical variables). We derived the cut-offs for *U2AF1* VAF by using Receiver Operating Characteristics (ROC) analysis to assess values that correlated with OS. The Kaplan–Meier method was used to estimate overall survival (OS). All tests were two-sided with *P* value < 0.05 considered statistically significant. Cox proportional hazards regression model was used to determine the univariate and multivariate predictors of overall survival in patients with high-risk myeloid neoplasm. Multivariable models included all significant univariate predictors with *P* = ≤0.05. We also performed landmark analysis for OS among responding patients from time of response till last follow up or death and evaluated the impact of allogeneic stem cell transplantation (allo-HCT) in these patients.

## Results

### Baseline characteristics

The baseline characteristics for this cohort are summarized in Table 1. Overall, the median age of the cohort was 72 years (range, 19–92), with a male preponderance (83%), and similar distributions in patients with CCUS, MDS, MDS/AML and AML. Twenty-two (12%), 108 (60%), 18 (10%) and 31 (17%) patients met criteria for CCUS, MDS, MDS/AML and AML, respectively. There was no significant difference in median white blood cell (WBC) count (*P* = 0.49), hemoglobin (*P* = 0.23) and platelets (*P* = 0.62) between CCUS, MDS, MDS/AML and AML patients. Sixty-seven % (*n* = 118) of these patients had cytogenetic (CG) abnormalities; CCUS (*n* = 17 [77%]), MDS (*n* = 70 [65%]), MDS/AML (*n* = 12 [70%]) and AML (*n* = 19 [61%]), *P* = 0.43. Overall, the most common CG abnormalities were del 20q (18%), complex CG (12%), trisomy 8 (9%) and monosomy 7/del 7q (8%) as outlined in Table 1. In MDS patients, 23% (*n* = 28), 54% (*n* = 64), and 23% (*n* = 28) had very good/good, intermediate risk and poor/very poor risk CG, respectively, as per IPSS-R (Revised International Prognostic Scoring System) criteria [22]. In the AML group, 20/31 (64.5%) had CG abnormalities, 50% (*n* = 10) each had ELN 2022 intermediate and adverse risk CG [7].

**Table 1:** Baseline characteristics of patients with *U2AF1* mutation; *N* = 179 (%)/[range].

Variable	Total *N* = 179	CCUS *N* = 22	MDS *N* = 108	MDS/AML *N* = 18	AML *N* = 31	*P* value
Age (years)	72 (19–92)	72 [56–92]	72 [50–92]	71 [19–90]	70 [19–90]	0.77
Age ≥70 years	73 (41.5)	9 (43)	42 (39)	7 (39)	15 (50)	0.76
Gender (male)	149 (83)	17 (81)	93 (86)	15 (83)	23 (74)	0.41
*U2AF1* mutation						
S34	107 (60)	12 (45)	62 (57)	11 (61)	17 (55)	0.17
Q157	63 (35)	10 (55)	42 (39)	6 (33)	11 (35)	0.36
Other variants	9 (5)	0	4 (4)	1 (6)	3 (10)	0.09
* U2AF1* VAF % median (range)	35 [5–51]	33 [6–51]	36 [5–49]	33 [5–44]	35 [10–46]	0.77
MDS (*n* = 108)						N/A
IPSS-R low risk	40 (37)	–	40 (37)	–		
IPSS-R intermediate risk	36 (33)	–	36 (33)	–		
IPSS-R high risk	21 (19)	–	21 (19)	–		
IPSS-R very high risk	11 (10)	–	11 (10)	–		
Therapy-related myeloid neoplasm	33 (15)	1	21 (17)	3 (11)	8 (23)	0.21
WBC (10^9^/L)	3 [0.40–116]	3.1 [1.1–8.8]	2.8 [0.8–53.3]	2.0 [0.4–8.3]	4.4 [0.5–116]	0.49
Hemoglobin (g/dl)	9 [5.8-14.9]	9.1 [6.2–13]	9.1 [6.2–14.9]	8.9 [5.8–13.1]	8.7 [6.9–14.1]	0.23
Platelet (10^9^/L)	85.5 [5–826]	161 [33–309]	85 [5–826]	108 [8–354]	49.5 [12–335]	0.62
BM blast (%)	3.8 [0–90]	0 [0–5]	2 [0–9]	11 [11–19]	43 [20–90]	0.004
Concurrent CG abnormality	118 (67)	17 (77)	70 (65)	12 (70)	19 (61)	0.43
del 20q	32 (18)	4 (18)	26 (24)	1 (5)	1 (3)	0.02
Complex CG*	22 (12)	1 (4.5)	14 (13)	1 (5)	6 (19)	0.26
Trisomy 8*	17 (9)	4 (18)	6 (6)	1 (6)	6 (19)	0.03
Monosomy 7/del 7q*	14 (8)	2 (9)	9 (8)	2 (11)	1 (3)	0.79
Other abnormalities	41 (23)	2 (9)	26 (24)	7 (39)	6 (19)	0.17
Myeloid co-mutations	144 (81)	17 (81)	86 (80)	16 (89)	25 (81)	0.88
Concurrent myeloid mutation observed in >5% of cases						
* ASXL1*	66 (37)	8 (33)	36 (33)	7 (39)	14 (45)	0.67
* BCOR*	34 (19)	5 (24)	21 (19)	5 (28)	3 (10)	0.39
* RUNX1*	25 (14)	1 (5)	15 (14)	4 (23.5)	5 (16)	0.41
* TET2*	26 (15)	2 (9.5)	18 (17)	2 (11)	4 (13)	0.79
* DNMT3A*	18 (10)	1 (5)	10 (9)	4 (22)	3 (10)	0.43
* RAS*	15 (8)	2 (9.5)	8 (7)	0	5 (16)	0.21
* TP53*	14 (8)	2 (9.5)	6 (5)	1 (5)	5 (16)	0.46
* JAK2*	10 (5.5)	0	8 (7)	1 (5)	1 (3)	0.44
* SETBP1*	9 (5)	0	5 (5)	0	4 (13)	0.07
No. of patients that received treatment (%)/CR (%)	125 (70)/29 (23)	6 (27)/(0)	75 (69)/19 (25)	17 (94)/3 (18)	27 (87)/7 (26)	<0.001
HMA	66 (53)/8 (12)	2 (33)/0	48 (64)/ 6 (12.5)	10 (59)/0	6 (22)/2 (33)	–
HMA plus venetoclax	10 (6)/5 (50)	0	4 (5)/3 (75)	1 (6)/1 (100)	5 (18.5)/1 (20)	
Intensive chemotherapy	23 (18)/16 (70)	0	6 (8)/2 (33)	3 (18)/3 (100)	14 (52)/11 (79)	
Other low-intensity/supportive care therapy	25 (20)/(0)	4 (67)/0	17 (23)/0	3 (18)/0	1 (4)/0	
Allogeneic stem cell transplant	31 (18)	0	19 (17.5)	4 (22)	8 (26)	0.02

*IPSS-R* revised international prognostic scoring system, *HMA* hypomethylating agent, *CR* complete remission, *PR* partial remission, *HI* hematological improvement, *VAF* variant allele frequency.

Low-intensity therapy (hydrea, growth factors, immunomodulators, low dose cytarabine)

*While the ICC includes these as MDS defining CG abnormalities, given that there is consensus needed between the WHO and ICC, for now we have not included these in the MDS category, as long as they did not meet criteria for morphological dysplasia.

### Somatic mutational profile and co-mutational patterns

**Fig. 1:**
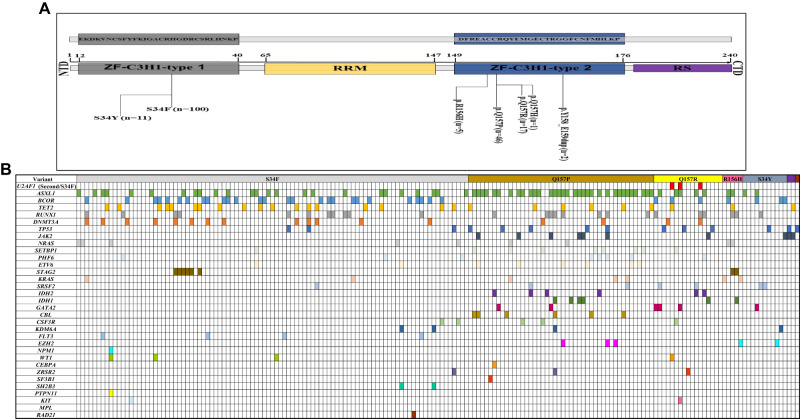
U2AF1 mutation structure and somatic co-mutation pattern. **a** Overview of *U2AF1* domains, structures, and distribution of *U2AF1* mutations detected, positioned on the *U2AF1* protein. Protein Sequence of ZF1 (hotspots at codon 34; S34F and S34Y) and ZF2 (hotspots at codon 157; Q157R and Q157P) domains, where all *U2AF1* mutations clustered. **b** Patterns of the co-mutations identified in the *U2AF1* cohort with respective mutations. NTD N-terminal domain, ZF zinc finger domain, RRM RNA recognition motifs, RS The C-terminal Arg-Ser rich domain, CTD C-terminal domain.

The median *U2AF1* variant allele frequency (VAF) was 35% (range [R], 5–51). The median *U2AF1* VAF was 33% (R, 6–51), 36% (R, 5–49), 33% (R, 5–44) and 35% [R, 10–46] in patients with CCUS, MDS, MDS/AML and AML, respectively (*P* = 0.77). *U2AF1* MT locations included S34 (60%), Q157 (35%), and others (5%). The corresponding mutational frequencies were 45%, 55%, and 0% in CCUS, 57%, 39%, and 4% in MDS, 61%, 33%, and 6%, in MDS/AML, 55%, 35% and 10% in AML (*P* = 0.17, *P* = 0.36 and *P* = 0.09, respectively). In the U2AF1 protein the S34 hotspot is in the zinc finger protein 1 region (ZF1), while the Q157 hotspot is located in the ZF2 region (Fig. 1). Concurrent myeloid MT observed in ≥ 5% of patients were *ASXL1* (37%), *BCOR* (19%), *RUNX1* (14%), *TET2* (15%), *DNMT3A* (10%), *RAS* (*NRAS* or *KRAS*) (8%), *TP53* (8%; 9/14 *TP53* MT were “multi-hit” as per ICC 2022 [accompanying CG or loss of heterozygosity, multiple *TP53* MT, single *TP53* MT with VAF > 50% or 17p deletion]), *JAK2* (5.5%) and *SETBP1* (5%), respectively. We did not observe co-occurrence of splicing factor *(SRSF2, SF3B1* and *ZRSR2)* MT with *U2AF1* MT. Illustrations depicting concurrent myeloid co-mutations with *U2AF1* MT and their corresponding VAF % are provided in Supplementary Figs. 1–3.

We analyzed myeloid co-mutation patterns between the two most frequent *U2AF1* MT (S34F and Q157P) (Table 2). Concurrent MT were more likely to be seen with Q157P compared to S34F (91% vs 74%; *P* = 0.01) MT, respectively. Cytogenetic abnormalities were more frequently seen with S34F compared to Q157P (70% vs 62%; *P* = 0.05). *U2AF1* S34F MT clustered with *BCOR* (*P* = 0.04) MT, while Q157P MT clustered with *ASXL1* (*P* = 0.01) and *TP53* (*P* = 0.03) MT (*P* = 0.02), respectively. We did not observe significant differences in median WBC (*P* = 0.23), hemoglobin (*P* = 0.82) and platelet counts (*P* = 0.72) between S34F and Q157P MT. We then looked at differences in hemoglobin level ≤10 g/dl (*P* = 0.16) or ≤8 g/dl (*P* = 0.51) between S34F and Q157P *U2AF1* MT and did not find statistically significant differences. Similarly, there were no differences in platelet counts between the two groups.

**Table 2:** Comparison of baseline characteristics among common U2AF1 mutations (*N* = 143).

Variables	S34F (*n* = 97)	Q157P (*n* = 46)	*P* value
Age	71 [25–92]	71 [53–92]	0.95
Age ≥70 years	56 (60)	26 (56.5)	0.85
High-risk myeloid neoplasm	26 (27)	13 (28)	>0.99
t-AML	17 (18)	7 (16)	0.81
CCUS	16 (16)	2 (4)	0.77
MDS	55 (57)	31 (67)	0.27
MDS/AML	10 (10)	4 (9)	0.77
AML	16 (17)	9 (20)	0.81
Hemoglobin (g/dl)	9.3 [6.4–13.3]	8.3 [5.8–13.1]	0.82
≤ 10.0	66 (69)	37 (80)	0.16
≤ 8.0	19 (20)	12 (26)	0.51
WBC (10^9^/L)	2.4 [0.5–10.3]	2.9 [0.9–60]	0.23
Platelet (10^9^/L)	93 [11–309]	83 [12–542]	0.72
≤ 100.0	51 (54)	24 (54.4)	>0.99
≤ 50.0	19 (20)	14 (32)	0.19
BM blast (%)	3 [0–83]	3.5 [0–90]	0.91
*U2AF1* VAF (%)	33 [2–51]	38 [11–46]	0.37
CG abnormality	68 (70)	28 (62)	**0.05**
Co-mutation	71 (74)	42 (91)	**0.01**
* ASXL1*	27 (28)	23 (50)	**0.01**
* BCOR*	22 (23)	0	**0.04**
* RUNX1*	11 (12)	9 (19)	0.46
* TET2*	14 (15)	5 (11)	0.60
* DNMT3A*	12 (12)	4 (9)	0.58
* RAS*	8 (8)	2 (4)	0.50
* TP53*	4 (4)	7 (15)	**0.03**
* JAK2*	4 (4)	5 (11)	0.27
* SETBP1*	5 (5)	4 (9)	0.47
* NPM1*	1 (1)	0	>0.99
* FLT3 ITD*	2 (2)	0	0.55
Two or more co-mutations	45 (47)	34 (73)	**0.02**
MDS patients progressing to AML	20/67 (30)	15/29 (52)	0.51
Treatment in high-risk myeloid neoplasm			
3 + 7	14 (20)	4 (13)	0.41
HMA	41 (58)	15 (33)	0.09
HMA plus venetoclax	4 (5.5)	5 (16)	0.72
Complete remission	19 (19.5)	7 (15)	>0.99
Allo-HCT	18 (19)	9 (21)	0.81

*t-AML* therapy-related AML, *CCUS* clonal cytopenia of undetermined significance, *CG* cytogenetics, *VAF* variant allele frequency, *HMA* hypomethylating agent, *CR* complete remission, *allo-HCT* allogeneic hematopoietic stem cell transplantation.

Bold values show statistically significant *p* values.

### Treatment and responses

One hundred twenty-five (70%) patients received disease directed treatment. The number of patients in each group that received treatment included 6/22 (27%) patients with CCUS, 75/108 (69%) patients with MDS, 17/18 patients with MDS/AML (94%) and 27/31 (87%) patients with AML. Four of 31 (13%) AML patients did not receive leukemia directed therapy due to co-morbidities and advanced age. Two of six (33%) CCUS patients received hypomethylating agents (HMA=off label use) and the remaining 4/6 (67%) received supportive care (e.g., erythropoietin stimulating agents and/or growth factor support). Amongst MDS patients, 48 (64%) received HMA, 17 (23%) received supportive care, 6 (8%) received AML-like intensive chemotherapy and 4 (5%) patients received HMA plus venetoclax combination therapy. In the MDS/AML cohort, 10 (59%) patients received HMA, 3 (18%) received intensive chemotherapy, 3 (18%) patients other low-intensity/supportive care therapy and 1 (6%) patient received HMA plus venetoclax combination therapy. In the AML cohort 14 (52%) received intensive chemotherapy, 6 (22%) patients received HMA, 5 (18%) patients received HMA plus venetoclax combination therapy and 1 (4%) received other low-intensity/supportive care therapy (Table 1).

None of the patients treated in the CCUS group met criteria for an objective response (as adjudicated by MDS response criteria, given that CCUS response criteria do not exist). The complete remission [CR] rates were 25% in the MDS cohort, 18% in MDS/AML cohort and 26% in AML cohort. Details on responses with regards to diagnostic categories and types of therapies used summarized in Table 1. Overall, 31 (18%) patients underwent allo-HCT; 19 (17.5%) patients with MDS, 4 (22%) patients with MDS/AML and 8 (26%) patients with AML. Treatment patterns, responses and proportion of patients receiving allo-HCT were not significantly different amongst the two common *U2AF1* MT (S34F and Q157P) (Table 2).

### Survival outcomes

**Fig. 2:**
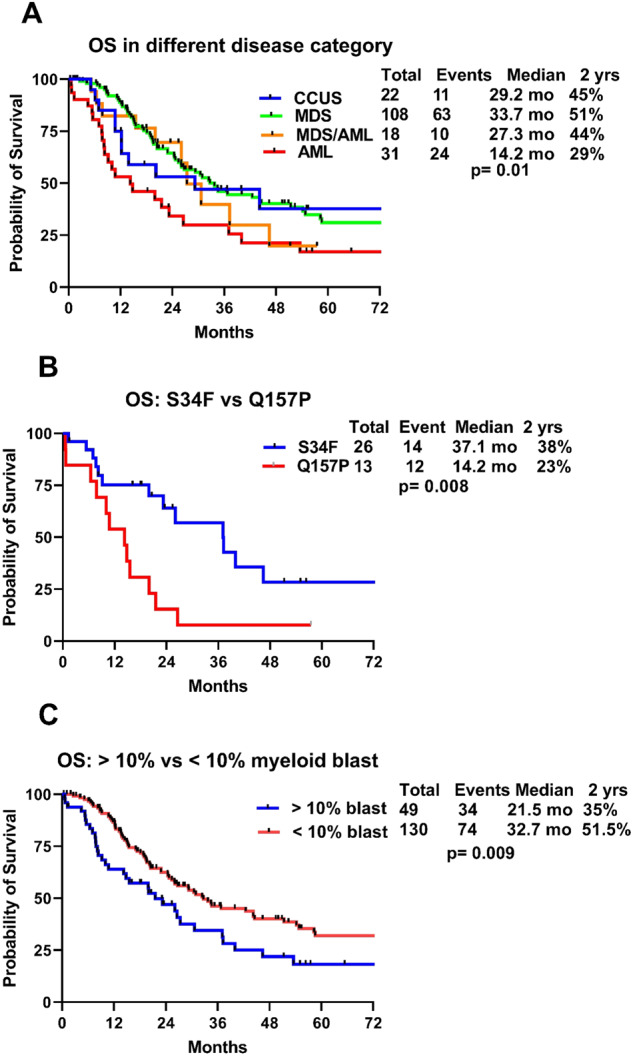
Kaplan–Meier overall survival curves. **a** In CCUS, MDS, MDS/AML and AML, **b** in high-risk myeloid neoplasm with S34F and Q157P mutations and **c** in relation to bone marrow blast <10% vs ≥10%.

**Fig. 3:**
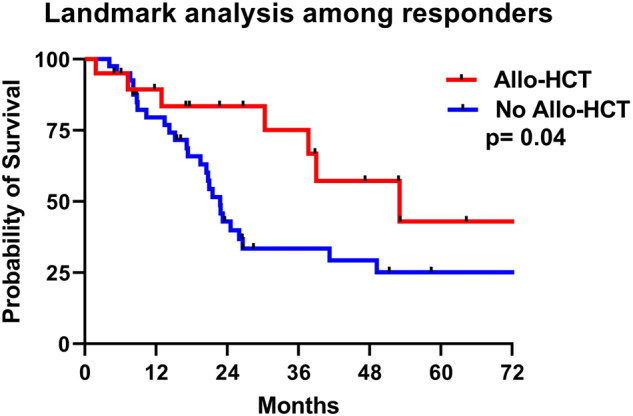
Landmark analysis for OS. Landmark analysis for overall survival among responding patients receiving allogeneic stem cell transplantation (allo-HCT).

The median OS of the entire cohort was 26.5 months. The median OS among patients with CCUS, MDS, MDS/AML and AML was 29.2, 33.7, 27.3 and 14.3 months, respectively (*P* = 0.01; Fig. 2a). The median OS in patients with high-risk myeloid neoplasm was 26.6 months. We then performed a subset analysis among patients with high-risk myeloid neoplasms harboring; S34F and Q157P MT. The median OS were 37.1 vs 14.2 months with *U2AF1*^S34F^ and *U2AF1*^Q157P^ MT, respectively (*P* = 0.008; Fig. 2b). The median OS was better in patients with <10% myeloid blasts compared to those with ≥10% myeloid blasts (32.7 vs 21.5 months, *P* = 0.009; Fig. 2c). We used ROC derived *U2AF1* VAF cut off for OS (VAF ≥ 25% vs <25%), however VAF as a continuous variable did not achieve statistical significance (52.3 vs 50.9 months, *P* = 0.48). Similarly, we also performed landmark analysis for OS among responding patients from time of response till last follow up or death and evaluated outcome with allo-HCT in these patients. The median OS was significantly better with allo-HCT (53.0 months) compared to without allo-HCT (22.8 months), *P* = 0.04 (Fig. 3).

### Univariate and multivariate analysis for OS in high-risk myeloid neoplasms with U2AF1^S34F^ or U2AF1^157P^

In univariate analysis for OS in patients with high-risk myeloid neoplasms*, U2AF1*^Q157P^ compared to *U2AF1*^S34F^ showed significantly inferior outcome (14.2 vs 37.1, *P* = 0.008). Patients with concurrent *ASXL1* MT (10.77 vs 37.1 months, *P* = 0.008), and *JAK2* MT (5.8 vs 23.2 months, *P* = 0.01) had inferior survival outcomes. Patients with bone marrow (BM) blast percentage ≥20% had inferior OS (14.3 months) compared to BM blast percentage between 10 and 19% (37.3 months, *P* = 0.03). Allo-HCT was associated with favorable OS in univariate analysis (40.0 vs 15.5 months, *P* = 0.04). Using predictors that demonstrated significance or trended towards significance (*P* = ≤ 0.1) in univariate analysis, a group of variables was assembled for multivariate OS analysis. Concurrent *JAK2* MT (HR: 8.12, 95% CI; 1.39–47.31, *P* = 0.02) and Q157P vs S34F (HR: 4.37, 95% CI; 1.31–14.11, *P* = 0.01) and retained significance for inferior OS, while allo-HCT (HR: 0.71, 95% CI; 0.09–0.57, *P* = 0.01) retained significance for better OS in multivariate analysis (Table 3).

**Table 3:** Univariate and multivariate analysis for overall survival in patient with high-risk myeloid neoplasm with S34F and Q157P mutations (*n* = 39).

Variables	Univariate analysis			Multivariate analysis		
	Hazard ratio	95% CI	*P* value	Hazard ratio	95% CI	*P* value
Age ≥70 years	1.82	0.82–4.0	0.11	–	–	–
BM blast ≥ 20% vs 10–19%	2.31	1.04–5.11	**0.03**	1.39	0.58–3.35	0.45
t-AML	0.75	0.30–1.81	0.37			
*U2AF1* mutations: Q157P vs S34F	2.64	1.08–6.47	**0.008**	4.37	1.31–14.11	**0.01**
CG abnormality	1.37	0.59–3.15	0.46	–	-	-
* ASXL1*	2.69	1.05–6.86	**0.008**	0.49	0.15–1.56	0.23
* BCOR*	0.48	0.19–1.20	0.12	–	–	–
* RUNX1*	1.24	0.47–3.29	0.65	–	–	–
* TET2*	0.83	0.30–2.29	0.72	–	–	–
* DNMT3A*	0.49	0.30–1.69	0.72	–	–	–
* RAS*	0.88	0.35–2.20	0.80	–	–	–
* TP53*	1.06	0.31–3.65	0.91	–	–	–
* JAK2*	4.90	0.24–98.1	**0.01**	8.12	1.39–47.31	**0.02**
* SETBP1*	0.38	0.10–1.40	0.32	–	–	–
* FLT3 ITD*	2.47	0.42–14.46	0.11	–	–	–
Two or more co-mutations	0.97	0.48–1.97	0.94	–	–	–
Treatment in high-risk MDS/AML						
3 + 7	0.66	0.35–1.26	0.71	–	–	–
HMA	0.33	0.70–2.02	0.33	–	–	–
HMA plus venetoclax	1.31	0.56–3.07	0.82	–	**–**	–
Complete remission (CR/CRi)	0.77	0.38–1.55	0.47	–	–	–
Allo-HCT	0.41	0.19–0.92	**0.05**	0.71	0.09–0.57	**0.01**

*VAF* variant allele frequency, *HMA* hypomethylating agent, *CR* complete remission, *PR* partial remission, *HI* hematological improvement.

Bold values show statistically significant *p* values.

## Discussion

We present data on the molecular profile and survival outcomes of patients with precursor myeloid neoplasms and myeloid neoplasms harboring *U2AF1* MT. We observed distinct myeloid co-mutation profiles and survival outcomes associated with different mutant *U2AF1* hotspot regions and amino acid changes. The most frequently observed MT were S34 (60%), Q157 (35%), followed by others (5%), in alignment with prior published data [15]. In high-risk myeloid neoplasm patients, *U2AF1*^Q157P^ was associated with inferior outcome compared to *U2AF1*^S34F^. *U2AF1*^Q157P^ MT significantly clustered with *TP53* (15% vs 4%) and *ASXL1* (50% vs 28%) mutations and had a higher percentage of co-mutations (91% vs 74%), in comparison to *U2AF1*^S34F^ MT. *U2AF1*^S34F^ MT on the other hand significantly clustered with *BCOR* MT (23% vs 0%). Cytogenetic abnormalities were more commonly seen with *U2AF1*^S34F^ MT compared to *U2AF1*^Q157P^ MT. While the observation that *U2AF1*^S34F^ and *U2AF1*^Q157P^ are the most frequent *U2AF1* MT in myeloid neoplasms is not new [5, 23–25], our study is the first to demonstrate their unique co-mutational spectrum and differential prognostic effect.

In our cohort, aligned with most previous observations, we did not observe co-occurrence of other splicing factor MT with *U2AF1* MT [26]. It is believed that concomitant splicing factor MT in the same cell could be incompatible with survival of the cell. In the new risk stratification schema for MDS and AML, *U2AF1* MT are now considered high risk [7, 27]; given that 81% of patients in our cohort had concurrent myeloid MT, we asked the question as to whether or not these accompanying MT were acting as confounding factors, adversely influencing *U2AF1* MT related outcomes. In our analysis, the OS of the entire cohort including patients with CCUS, MDS, MDS/AML and AML was sub-optimal at 26.5 months, with a differential prognostic impact imparted by the two *U2AF1* hotspot regions; 37.1 vs 14.2 months (*P* = 0.008) in patients with high-risk myeloid neoplasm harboring *U2AF1*^S34F^ and *U2AF1*^Q157P^MT, respectively. Earlier reports suggested differential degree of anemia and thrombocytopenia with different *U2AF1* MT. In MDS, thrombocytopenia was specifically associated with *U2AF1*^*S34F*^ MT and anemia with *U2AF1*^*Q157*^ MT [24]. In patients with myelofibrosis, both mutation types were associated with anemia and the association with thrombocytopenia was most evident with *U2AF1*^*Q157*^ MT [10]. In our cohort of patients with CCUS, MDS, MDS/AML and AML, we did not observe significant differences in anemia or thrombocytopenia among different *U2AF1* MT. Interestingly, in the current study we observe a relatively higher rate of CG abnormalities and a shorter OS in *U2AF1* MT CCUS patients, in comparison to patients with MDS and AML. Our group has recently reported on clinical outcomes of patients with *U2AF1* MT clonal hematopoiesis (CHIP [clonal hematopoiesis of indeterminate potential] and CCUS) [16]. In that study, we observed a high rate (25%) and a short latency (17.5 months) towards progression to myeloid neoplasms. We acknowledge that the higher incidence of CG abnormalities in the CCUS group could have been due to selection bias, inherent to the structure of retrospective studies.

Current literature suggest sub-optimal responses with hypomethylating agent therapies in *U2AF1*-mutated myeloid neoplasms [28], and better responses with HMA plus venetoclax-based therapies [6]. Similarly, we observed lower CR rates with HMA therapy alone (12%) and relatively better responses with HMA plus venetoclax (45%) or intensive chemotherapies (65%). However, our sample size was small and larger prospective studies are needed to gauge responses with different treatment regimens. Similar to earlier reports, our study suggests benefit from allo-HCT in improving survival outcome among patients with *U2AF1* MT myeloid neoplasms [29].

We acknowledge the limitations of our analysis including inherent selection bias and lack of sequential mutation testing in this cohort of patients with myeloid neoplasms. Nevertheless, our study highlights the unique co-mutation patterns and survival outcomes in CCUS, MDS and AML patients with different *U2AF1* MT, underscoring the need for accurate mutational assessment and reporting.

### Supplementary information

Supplementary Figure 1

Supplementary Figure 2

Supplementary Figure 3

Supplementary Material

Supplementary figure legend

## Data Availability

The data that support the findings of this study are available from the corresponding author upon reasonable request.
