# Supplementary figures and images for: U2AF1 pathogenic variants in myeloid neoplasms and precursor states: distribution of co-mutations and prognostic heterogeneity
Source: Blood Cancer J. 2023 Sep 21;13(1):149. doi: 10.1038/s41408-023-00922-7 (PMC10514309; doi:10.1038/s41408-023-00922-7)

## Slide 1
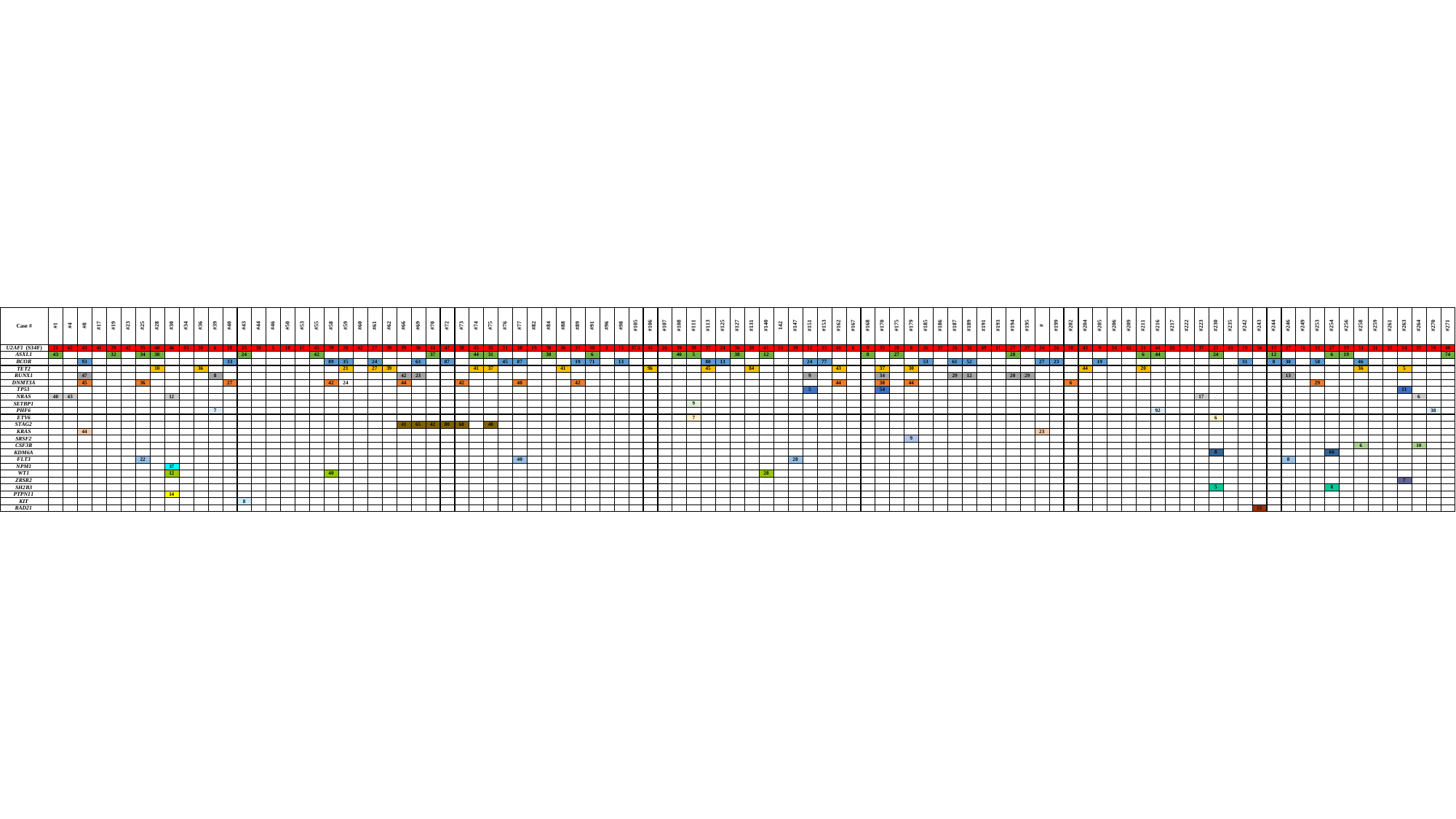

Supplement: Supplementary file 1 — Supplementary Figure 1 [file 41408_2023_922_MOESM1_ESM.pptx]

## Slide 1
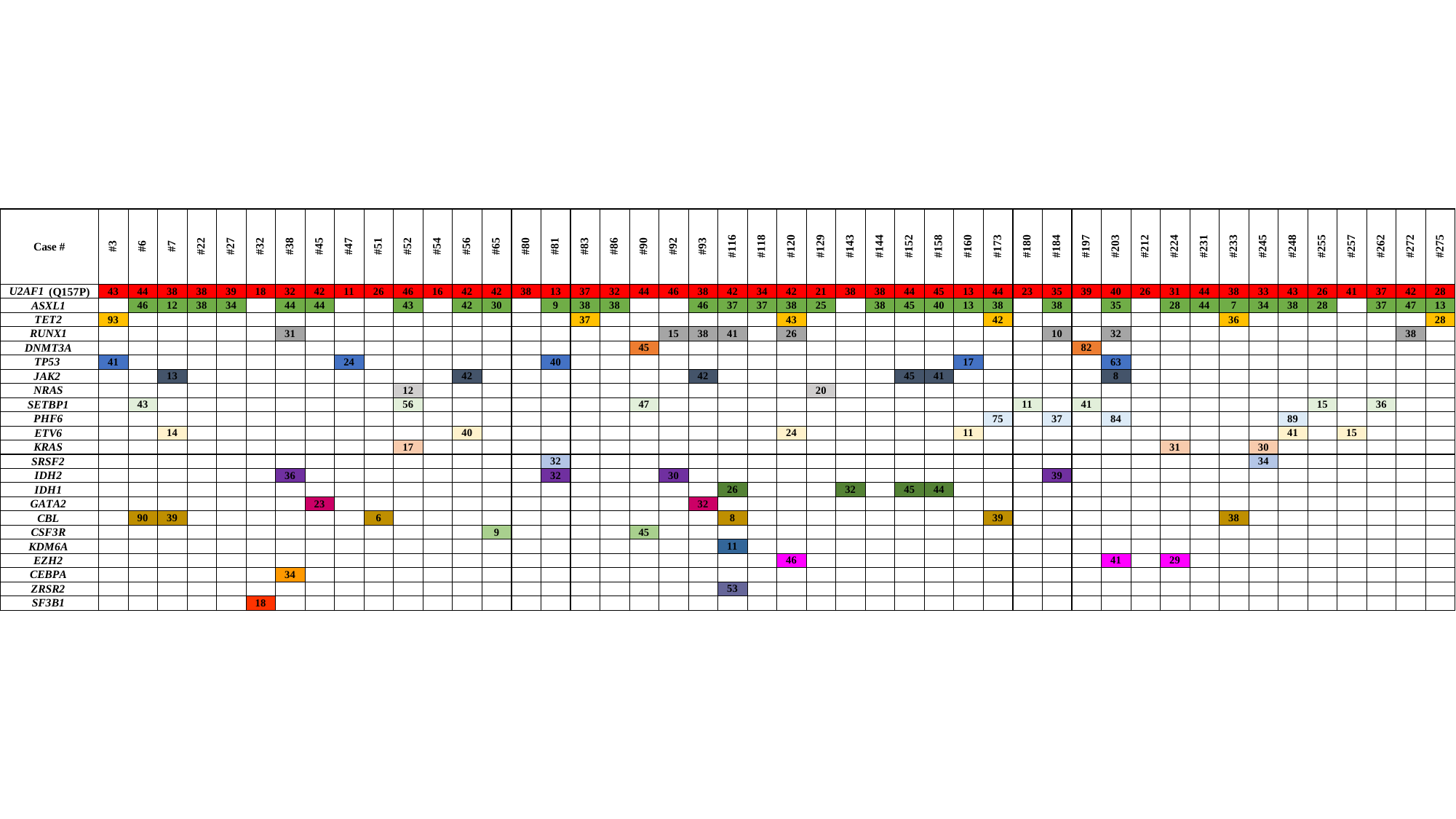

Supplement: Supplementary file 2 — Supplementary Figure 2 [file 41408_2023_922_MOESM2_ESM.pptx]

## Slide 1
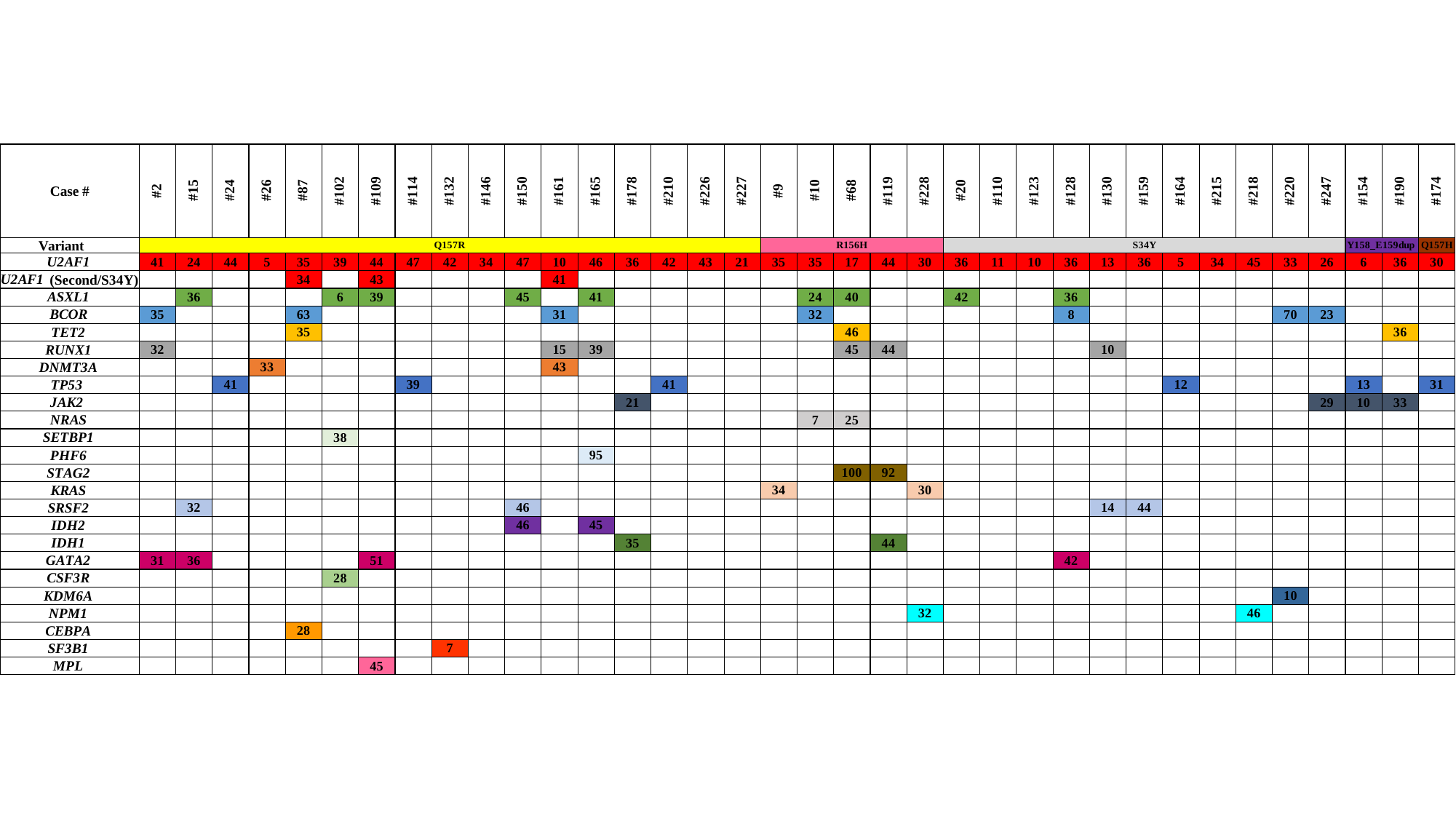

Supplement: Supplementary file 3 — Supplementary Figure 3 [file 41408_2023_922_MOESM3_ESM.pptx]
