# Supplementary material for: U2AF1 pathogenic variants in myeloid neoplasms and precursor states: distribution of co-mutations and prognostic heterogeneity
Source: Blood Cancer J. 2023 Sep 21;13(1):149. doi: 10.1038/s41408-023-00922-7 (PMC10514309; doi:10.1038/s41408-023-00922-7)
Supplement: Supplementary file 4 — Supplementary Material [file 41408_2023_922_MOESM4_ESM.docx]

**42 NGS panel at Mayo Clinic**

ANKRD26  (NM_014915.2) 5'UTR, exons 1-4, intron c.172,  ASXL1  (NM_015338.5)
exons 10-13,  BCOR  (NM_001123385.1) exons 4-15,  CALR  (NM_004343.3) exon 9,
CBL  (NM_005188.3) intron 7 last 100bps before start of exon 8, exon 8,
intron 8, and exon 9,  CEBPA  (NM_004364.4) exon 1,  CSF3R  (NM_000760.3)
exons 14 and 17,  DDX41  (NM_016222.2) exons 1-17,  DNMT3A  (NM_022552.4)
exons 8-23, ELANE  (NM_001972.2) exons 1-5,  ETNK1  (NM_018638.4) exons 2-5,
ETV6(NM_001987.4) exons 3-8,  EZH2  (NM_004456.4) exons 2-20,  FLT3
(NM_004119.2) exons 14-20,  GATA1  (NM_002049.3) exons 2 and 4,  GATA2
(NM_001145661.1) exons 3-7, intron 5, c.1017+1 - 1017+730,  IDH1
(NM_005896.3) exon 4,  IDH2(NM_002168.3) exon 4,  JAK2  (NM_004972.3) exons
12-16,  KDM6A  (UTX) (NM_021140.3) exons 1-29,  KIT  (NM_000222.2) exons 8-11
and 17,  KRAS(NM_033360.3) exons 2-3,  MPL  (NM_005373.2) exons 10-12,
NPM1(NM_002520.6) exons 9-11, to -30 before exon 11,  NRAS  (NM_002524.4)
exons 2 and 3,  PHF6  (NM_001015877.1) exons 2-10,  PTPN11  (NM_002834.3)
exons 3-4 and 12-13,  RAD21  (NM_006265.2) exons 1, 2, 4-7, 9-11, 13, 14,
exon 10 flank 15bp,  RUNX1  (NM_001001890.2) exons 1-6, intron 4 c.725-13T>A
and intron 5 c.886+1-4del,  SETBP1  (NM_015559.2) partial exon 4; amino acids
400 - 950, SH2B3  (LNK) (NM_005475.2) exon 2-8,  SF3B1  (NM_012433.2) exons
13-16, SRP72  (NM_006947.3) exons 6, 10,  SMC3  (NM_005445.3) exons 7, 8, 13,
17, 19, 21, 29,  SRSF2  (NM_003016.4) exons 1 and 2,  STAG2  (NM_001042750.1)
exons 4-34, 12, 17 and 22 flank 15bp,  TERT  (NM_198253.2) exons 2-16,
TET2(NM_001127208.2) exons 3-11,  TP53  (NM_000546.4) exons 4-9,
U2AF1(NM_001025203.1) exons 2, 6, and 8,  WT1  (NM_024426.2) exons 1-10, and
ZRSR2  (NM_005089.3) exons 1-11.
