# Supplementary material for: U2AF1 pathogenic variants in myeloid neoplasms and precursor states: distribution of co-mutations and prognostic heterogeneity
Source: Blood Cancer J. 2023 Sep 21;13(1):149. doi: 10.1038/s41408-023-00922-7 (PMC10514309; doi:10.1038/s41408-023-00922-7)
Supplement: Supplementary file 5 — Supplementary figure legend [file 41408_2023_922_MOESM5_ESM.docx]

**Supplementary Figure Legend**

**Supplementary figure 1**. Illustrations depicting concurrent myeloid co-mutations with *U2AF1* S34F MT and their corresponding VAF %.

**Supplementary figure 2**. Illustrations depicting concurrent myeloid co-mutations with *U2AF1* Q157P MT and their corresponding VAF %.

**Supplementary figure 3**. Illustrations depicting concurrent myeloid co-mutations with *U2AF1* Q157R/R156H/S34Y MT and their corresponding VAF %.
